# Differential Protein Expression in Human Dental Pulp: Comparison of Healthy, Inflamed, and Traumatic Pulp

**DOI:** 10.3390/jcm8081234

**Published:** 2019-08-16

**Authors:** Wonyoung Yue, Sunil Kim, Han-Sung Jung, Jong-Min Lee, Sukjoon Lee, Euiseong Kim

**Affiliations:** 1 Microscope Center, Department of Conservative Dentistry and Oral Science Research Center, College of Dentistry, Yonsei University, Seoul 03722, Korea; 2 Division in Anatomy and Developmental Biology, Department of Oral Biology, Oral Science Research Center, BK21 PLUS Project, Yonsei University College of Dentistry, Seoul 03722, Korea; 3 Department of Applied Life Science, BK21 PLUS Project, Yonsei University College of Dentistry, Seoul 03722, Korea

**Keywords:** dental pulp, dental pulp inflammation, proteome, dentin-pulp complex regeneration, annexin V, mass spectrometry

## Abstract

Trauma or injury to the dental pulp causes inflammation. This study compared the proteome of healthy pulp with inflamed pulp and traumatic pulp to identify the differentially expressed proteins in the diseased state. Five participants were grouped based on the pulpal status of the teeth: healthy, inflamed, or traumatic pulp. Pulp was extirpated and stored immediately in liquid nitrogen. Pulp tissues were subjected to 2-dimensional gel electrophoresis, and spot selection was performed. The selected spots were analyzed using liquid chromatography-tandem mass spectrometry and identified by correlating mass spectra to the proteomic databases. Fifteen spots showed increased expression in the inflamed and traumatic pulp. Annexin V, type II keratin, and hemoglobin levels were increased two-fold in the inflamed and traumatic pulp group and annexin V, mutant beta-actin, and hemoglobin were increased by ten-fold in the inflamed or traumatic pulp group, compared to levels in the healthy pulp group. Annexin V constituted two out of fifteen protein spots, and seemed to play a critical role in inhibiting inflammation and promoting the immune reaction. Further studies on this protein concerning its role in pulp repair are necessary to elucidate the underlying mechanisms.

## 1. Introduction

The dental pulp consists of various cell types, each with specified roles. The primary function of the dental pulp is to form dentin via odontoblasts. It also has nutritive, sensory, protective, and other formative functions [1]. Following dental tissue damage, initiation of an inflammatory response known as pulpitis occurs. After removal of the damage causative factor, the dental pulp will return to normal. However, if damage persists, the pulpitis becomes irreversible and leads to necrosis [2].

Pulpal repair in response to dental tissue damage caused by caries or trauma is a complex inflammatory process [3]. As caries progresses from the tooth surface toward the dentin-pulp complex, activation of both the innate and adaptive immunities occurs [4]. For a relatively mild stimulus, such as slowly progressing caries or tooth wear, the dentin-pulpal complex produces tertiary dentin. In contrast, under conditions of more rapidly progressing disease, a more intense immune and inflammatory response occurs, resulting in pulp tissue death [5]. During healing after dental tissue injury, induction of necrosis of pulp tissue, including odontoblasts, occurs followed by reparative dentinogenesis, a result of continuous and interrelated processes, including chemotaxis, proliferation, angiogenesis, extracellular matrix remodeling, and cell differentiation [6]. Wei et al. [6] reported that after tissue injury, the unique dentin-pulp complex might undergo complete regeneration, including the differentiation of various cell types and induction of new proteins. Smith et al. [7] reported the presence of a broad spectrum of bioactive molecules in the dentin-pulp complex. Eckhardt et al. [8] pointed out that identifying the bioactive proteins may facilitate a better understanding of their potential involvement in regenerative and other tissue responses. Therefore, elucidation of differences in the protein expression between healthy and diseased pulp tissue may help assess whether these differences offer novel clinical therapeutic methods [7,8].

Several new methods for evaluation of protein expression have been developed. Proteomic approaches using two-dimensional gel electrophoresis (2-DE), coupled with protein identification by mass spectrometry (MS) and bioinformatics, offer a high-throughput means to study different protein expression profiles relating to a particular pathophysiological condition [6]. Proteomic techniques are now widely accepted as an important tool for identifying potential biomarkers and studying the mechanisms involved in cell differentiation [9].

Several reports showed the use of proteomic analysis for investigation of the human dental pulp and most studies analyzed in vitro or ex vivo cultured cells. Very few studies analyzed human dental pulp itself [8,10,11]. Paakkonen et al. [10] identified 96 proteins in pulp from healthy and carious human teeth, and Eckhardt et al. [8] detected 342 pulp proteins, including 37 proteins not previously discovered in human dentin or plasma. Jágr et al. [11] compared pulp proteins of sound tooth obtained from caries-resistant versus caries-susceptible individuals and showed that the levels of 16 proteins were significantly different in the caries-resistant group, compared to the susceptible individuals.

To the best of our knowledge, no reports exist on proteomic studies comparing healthy pulp with inflamed pulp and/or traumatic pulp. Therefore, the purposes of this study were to (1) compare the proteins present in healthy pulp with those in the inflamed pulp or traumatic pulp and (2) identify differentially expressed proteins during disease using 2-DE followed by capillary liquid chromatography-tandem mass spectrometry (LC-MS/MS) analysis.

## 2. Experimental Section

### 2.1. Sample Collection and Preparation

Approval for this project was obtained from the Yonsei University Committee for Research on Human Subjects (2-2015-0050), and informed consent was acquired from all participants. All extraction and preparation procedures were performed by a single operator at the Department of Conservative Dentistry, Yonsei University Dental Hospital, Seoul, Republic of Korea. Five participants aged 22–35 years (three males and two females) were enrolled into three groups based on the pulpal status of the tooth being treated. The groups were as follows:

1. Healthy pulp (*n* = 1; 22/M): two healthy and completely erupted permanent human third molars with closed apex, which were caries free showing normal probing depths.
2. Inflamed pulp group (*n* = 2; 23/F and 35/M): maxillary central incisors with advanced dental caries, defined as the presence of spontaneous pain and lingering response to the cold test.
3. Traumatic pulp group (*n* = 2; 24/F and 35/M): maxillary central incisors with closed apex, ten days after replantation due to traumatic avulsion and avulsed time within 20 min.

After obtaining informed consent, local anesthesia with lidocaine hydrochloride (2% with epinephrine 1:100,000) was administered. For healthy teeth, the teeth were extracted, and the root canal system was immediately accessed. The pulp was extirpated using a sterilized Hedstrom hand file (Dentsply Maillefer, Ballaigues, Switzerland). For teeth diagnosed with advanced dental caries or traumatic pulp, rubber dam isolation was performed; after obtaining access, pulp tissue was collected using a sterilized Hedstrom hand file. The pieces of pulp were lyophilized and frozen in liquid nitrogen. Each pulp sample was adjusted to about 320 µg dry weight.

### 2.2. Two-Dimensional Electrophoresis

2-DE was carried out as described previously [12]. Aliquots in sample buffer (7 mol/L urea, 2 mol/L thiourea, 4.5% 3-[(3-cholamidopropyl) dimethylammonio)-1-propanesulfonatehydrate, 100 mmol/L 1,4-dithioerythritol, 40 mmol/L Tris; pH = 8.8) were applied to immobilized pH 3–10 nonlinear gradient strips (Amersham Biosciences, Uppsala, Sweden). Isoelectric focusing was performed at 80,000 voltage hours. The second dimension was analyzed on 9–16% linear gradient polyacrylamide gels (18 cm × 20 cm × 1.5 mm) at a constant 40 mA per gel for approximately 5 h. After protein fixation in 40% methanol and 5% phosphoric acid for 1 h, the gels were stained with Coomassie Brilliant G-250 for 12 h. The gels were destained with H_2_O, scanned in a densitometer (GS710; Bio-Rad Laboratories, Richmond, CA, USA), and the obtained data were converted into electronic files, followed by analysis with the Image Master Platinum 5.0 image analysis program (Amersham Biosciences, Little Charfent, UK).

### 2.3. Spot Selection for Identification

It was hypothesized that some bioactive proteins would be over-expressed under conditions of stress to promote healing of the pulp tissue. Therefore, the following conditions were determined discretionally, and the spots corresponding to these conditions were selected.

1. Spots in the inflamed and traumatic pulp group with an intensity greater than 2.0 times that in the healthy pulp group
2. Spots in the inflamed or traumatic pulp group with an intensity greater than 10.0 times that in the healthy pulp group

### 2.4. In-Gel Tryptic Digestion

Spots of interest were excised from the preparative gel and transferred to individual 1.5-mL tubes. The gel sample was washed with 100 μL of distilled water; and 100 μL of 50 mmol/L NH4HCO3 (pH 7.8) and acetonitrile (6:4) were added and agitated for 10 min. This process was repeated at least three times until the Coomassie brilliant blue G250 dye had disappeared. After decanting the supernatant, the gel was dried in a speed vacuum concentrator (LaBoGeneAps, Lynge, Denmark) for 10 min, followed by digestion with sequence-grade modified trypsin (Promega Co., Madison, WI, USA) (enzyme to substrate ratio = 1:30) at 37 °C with shaking for 16 h.

### 2.5. LC-MS/MS for Peptide Analysis

Nano LC-MS/MS analysis was performed using an Easy *n*-LC (Thermo Fisher, San Jose, CA, USA) and an LTQ Orbitrap XL mass spectrometer (Thermo Fisher) equipped with a nano-electrospray source. Samples were separated on a C18 nanobore column (150 mm × 0.1 mm, 3 μm pore size; Agilent, Santa Clara, CA, USA). The mobile phase A for LC separation consisted of 0.1% formic acid and 3% acetonitrile in deionized water, while the mobile phase B had 0.1% formic acid in acetonitrile. A linear chromatography gradient of 5% B to 30% B in 23 min, 30% B to 60% B in 3 min, 95% B in 3 min, and 3% B in 6 min with a flow rate of 1500 nL/min was used. Mass spectra were acquired using data-dependent acquisition with a full mass scan (350–1200 *m/z*) followed by 10 MS/MS scans. For MS1 full scans, the orbitrap resolution was 15,000 and the AGC was 2 × 105. For MS/MS in the LTQ, the AGC was 1 × 104.

### 2.6. Database Search

The mascot algorithm (Matrix Science, Boston, MA, USA) was used to identify the peptide sequences present in the protein sequence database. Database search criteria were as follows: taxonomy, Homo sapiens (downloaded 19 June 2015); fixed modification, carbamidomethylated (+57) at cysteine residues; variable modification, oxidized (+16) at methionine residues; maximum allowed missed cleavage, 2; MS tolerance, 10 ppm; and MS/MS tolerance, 0.8 Da. The peptides were filtered with a significance threshold of *p* < 0.05.

## 3. Results

### 3.1. 2-DE and Image Analysis

This section may be divided by subheadings. It should provide a concise and precise description of the experimental results, their interpretation as well as the experimental conclusions that can be drawn.

**Figure 1:**
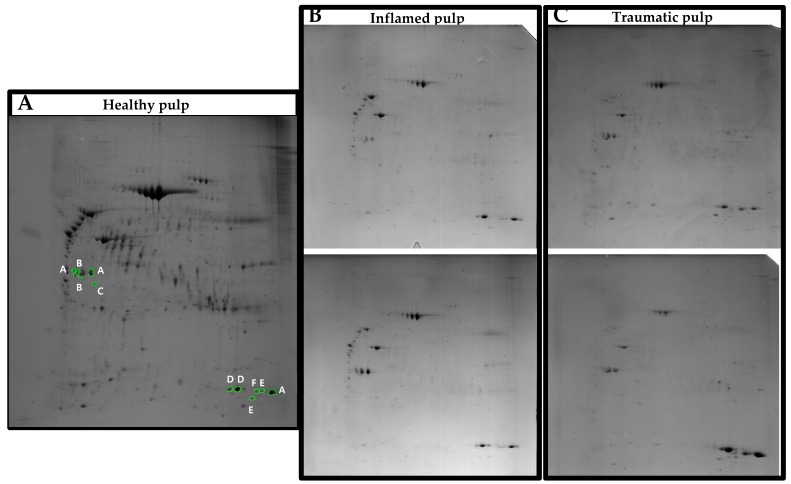
2-DE images of gels from three experimental groups. (**A**) Healthy pulp group. (**B**) Inflamed pulp group. (**C**) Traumatic pulp group. Green circles indicate the spots that were excised for mass spectrometry. A, type II keratin; B, annexin V chain A; C, mutant beta-actin; D, hemoglobin chain B; E, hemoglobin chain A; F, hemoglobin alpha 1-2 hybrid.

**Figure 2:**
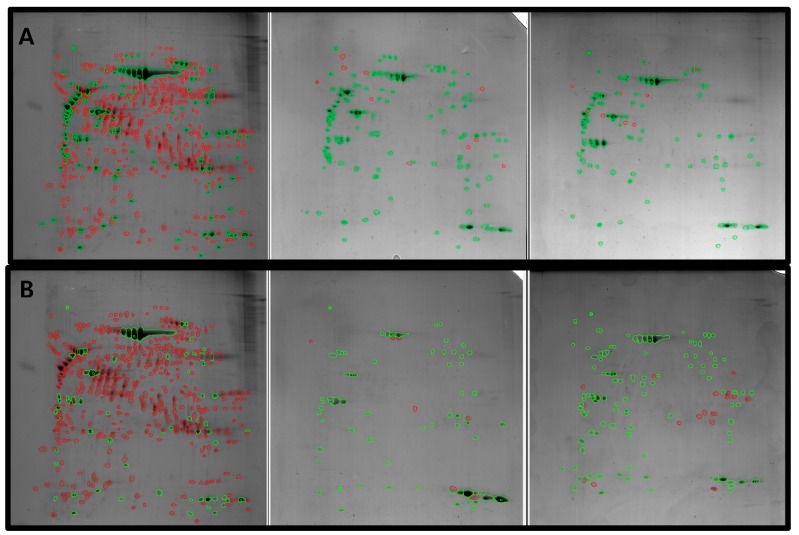
Paired 2-DE images. (green = paired spots/red = non-paired spots) (**A**) Healthy pulp and inflamed pulp groups. (**B**) Healthy pulp and traumatic pulp groups.

2-DE images were obtained for all three experimental groups (Figure 1). The overall spot patterns within the group were similar. About 530 spots in the healthy pulp, 135 and 124 spots in each inflamed pulp gel, and 100 and 73 spots in each traumatic pulp gel were detected. Hundreds of spots were absent in the experimental group gels, compared with the healthy pulp gel (Figure 2, red).

### 3.2. Spot Selection for Protein Identification

Fifteen spots were selected based on the conditions listed in the Material and Methods section. Six spots in the inflamed and traumatic pulp groups had an intensity that was more than two-fold higher, and nine spots in the inflamed or traumatic pulp groups had an intensity that was more than ten-fold higher than those in the healthy pulp group.

### 3.3. LC-MS/MS for Peptide Analysis

The six spots with higher than two-fold increase in both experimental groups represented annexin V, type II keratin and hemoglobin, and the nine spots with a higher than ten-fold increase in the experimental groups represented annexin V, mutant beta-actin, and hemoglobin. The protein identification results from the LC-MS/MS are listed in Table 1.

## 4. Discussion

Pulp tissue is mainly composed of connective tissue and also contains several other tissue types, including blood vessels and nerve fibers [10]. Pulp inflammation is responsible, as in other connective tissues, for the permanent loss of normal tissue function [13]. Necrosis is a passive process that occurs due to the loss of protein functions or plasma membrane integrity [14]. Consequently, the intensity and number of protein spots decreases as pulp inflammation progresses. However, it is reasonable to assume that a protein that plays an important role in pulp healing would be upregulated in the injured tissue. Hence, the spots with increased expression in the inflamed and traumatic pulp groups were selected and analyzed in this study.

When a tooth avulsion occurs, prognosis of the tooth is good when repositioned as soon as possible. Replantation should therefore be done immediately, but root canal treatment be postponed [15,16]. Endodontic literature suggested that, after 10 to 14 days of replantation, the tooth acquires sufficient stability to allow preparation of an access cavity for the root canal treatment because of partial repair of the injured periodontal ligament [17,18] and because inflammatory root resorption has just been initiated at that time [19]. Thus, in this study, we extirpated pulp tissue 10 days after replantation following the clinical practice protocols.

Hyperemia has long been recognized as a process of pulp inflammation [20,21,22]. As the pulp progresses from a healthy to an inflammatory state, inflammatory mediators stimulate vasodilation and increase microvascular permeability [23]. In one study, Pääkkönen et al. [10] found high expression of the placenta-derived growth factor (PIGF) gene in carious tissue. PIGF is a member of the vascular endothelial growth factor family, and the PIGF upregulation might promote cell migration and induce endothelial cell proliferation and permeabilization of blood vessels. Correspondingly, we noticed increased hemoglobin among the spots analyzed.

**Figure 3:**
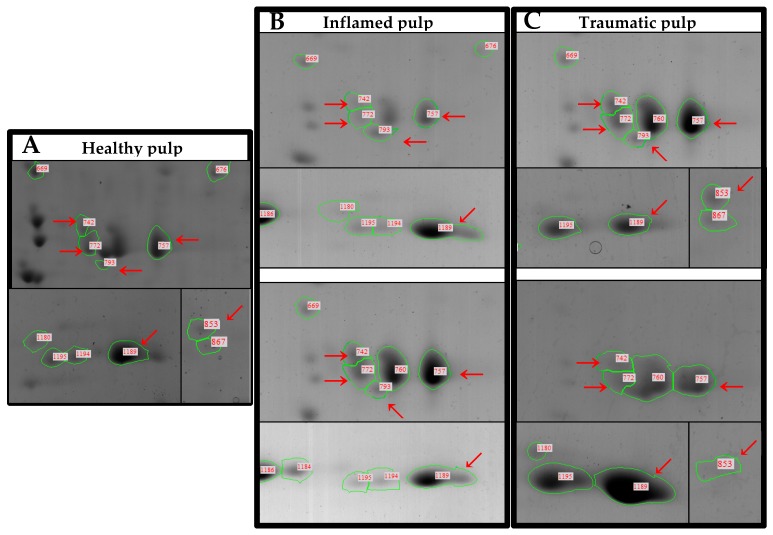
Closed 2-DE images of gels from the three experimental groups. (**A**) Healthy pulp group. (**B**) Inflamed pulp group. (**C**) Traumatic pulp group. Red arrows: spots with a greater than ten-fold increase in intensity, compared to those in the healthy pulp group. 742, 757 and 1189, type II keratin; 772 and 793, annexin V chain A; 853, mutant beta-actin.

Annexin V is a cellular protein in the annexin group that plays a central role in the process of cell membrane repair via the formation of a two-dimensional protective bandage at sites of membrane damage [11]. Current knowledge of cell membrane repair and findings on the role of annexin V in membrane resealing were reviewed recently [24]. Liu et al. [25] proposed that annexin V inhibits the inflammatory effect of phospholipids, decreases vascular inflammation, and promotes the induction of regulatory T cells and thus is potentially attractive as a therapeutic agent. Jágr et al. [11] reported increased annexin V expression in caries-resistant subjects compared to caries-susceptible subjects. These results support the findings in the present study, where annexin V constituted two out of the fifteen analyzed protein spots (Figure 3, red arrows: 772 and 793).

Actins are highly conserved proteins [26] involved in cell motility, structure, and integrity. Beta-actin (human gene and protein symbol ACTB/ACTB), one of the two non-muscle cytoskeletal actin isoforms, is one of six different actin isoforms which have been identified in humans. In this study, mutant beta-actin expression was increased ten-fold in the experimental groups, compared to the healthy pulp (Figure 3, red arrow: 853). Jágr et al. [11] reported an upregulation of the actin cytoskeleton component, tropomyosin alpha-1 chain protein, in caries-resistant teeth, but further research is necessary to verify the contributions of beta-actin.

Type II keratin was the third protein found to be upregulated by more than two-fold in the injured pulp (Figure 3, red arrows: 742, 757, and 1189). Keratin is the most common contaminant in many mass spec labs because it is present everywhere, on our hands and hair. Hodge et al. [27] reported that keratin could also be introduced from unexpected sources because type II keratin is present in all mammalian epithelial cells.

## 5. Conclusions

In 2014, the proteome map of human tooth pulp was reported, with a total of 342 proteins identified [8]. In the present study, we compared the proteomic profiles of healthy pulp with inflamed and traumatic pulp. This study improves the current understanding of the difference between healthy pulp and inflamed or traumatic pulp to a limited extent. Specifically, annexin V seems to play a critical role in the inhibition of inflammation and promotion of the immune reaction. Further studies are needed to better understand the role of annexin V in pulp inflammation.

## Figures and Tables

**Table 1:** Identity of selected spots using liquid chromatography-tandem mass spectrometry (LC-MS/MS) analysis.

Spot ID	Protein Name	Protein Score	Peptide Matched	Coverage (%)	Healthy Pulp (Vol.%)	Inflamed Pulp1 (Vol.%)	Inflamed Pulp2 (Vol.%)	Traumatic Pulp1 (Vol.%)	Traumatic Pulp2 (Vol.%)
742	keratin, type II	325	11	12	0.080	0.162	0.799	0.518	0.617
757	keratin, type II	449	13	12	0.480	1.141	4.742	2.024	3.532
772	annexin V chain A	194	7	13	0.156	0.412	1.450	0.657	0.632
1186	hemoglobin chain B	649	32	61	0.905	5.430	5.675	5.584	16.633
1189	keratin, type II	526	13	16	0.839	9.564	10.279	4.387	22.620
1195	hemoglobin alpha 1-2 hybrid	449	22	66	0.110	0.771	1.249	3.508	8.119
554	gelsolin isoform a precursor	152	4	3	0.028			* **0.328**	* **0.300**
793	annexin V chain A	739	20	39	0.030	0.401		* **1.274**	
853	mutant beta-actin	193	7	13	0.043			* **0.448**	0.373
1062	unnamed protein product	744	25	20	0.059			* **1.725**	* **0.897**
1083	unnamed protein product	866	27	26	0.025			* **1.124**	0.174
1183	unnamed protein product	441	15	16	0.096			* **1.471**	0.301
1188	hemoglobin chain B	469	24	75	0.306	1.228	1.303	1.457	* **4.523**
1194	hemoglobin chain A	257	10	31	0.088	* **0.808**	* **1.636**		
1217	hemoglobin chain A	259	11	42	0.102			0.402	* **1.049**

* indicates an increase of more than ten times that of healthy pulp. Database: NCBInr_human human_20150619 (182,730 sequences; 70,344,302 residues). Protein score is −10 *Log(P), where P is the probability that the observed match is a random event. Individual protein scores >26 indicate identity or extensive homology (*p* < 0.05).
